# Association between prediabetes (defined by HbA1_C_, fasting plasma glucose, and impaired glucose tolerance) and the development of chronic kidney disease: a 9-year prospective cohort study

**DOI:** 10.1186/s12882-019-1307-0

**Published:** 2019-04-16

**Authors:** Gwang Sil Kim, Hyun Ho Oh, Sang Hyun Kim, Byung Ok Kim, Young Sup Byun

**Affiliations:** 1 Division of Cardiology, Department of Internal Medicine, Sanggye Paik Hospital, Inje University, Dongil-ro 1342, Nowon-gu, Seoul 01757 South Korea; 2 Division of Nephrology, Department of Internal Medicine, Sanggye Paik Hospital, Inje University, Seoul, South Korea

**Keywords:** Prediabetes, Chronic kidney disease, Cardiovascular disease, General population

## Abstract

**Background:**

The aim of the present study was to investigate the clinical impact of prediabetes on the development of incident chronic kidney disease (CKD) in a Korean adult population, using data from the Korea Genome and Epidemiology Study.

**Methods:**

This prospective cohort study included 7728 Korean adults without baseline CKD and type 2 diabetes. Prediabetes was defined by impaired fasting glucose (IFG), impaired glucose tolerance (IGT), and HbA1_C_ level. CKD was defined as estimated glomerular filtration rate < 60 mL/min/1.73 m^2^. We assessed the predictive value of prediabetes for the incidence of CKD, and investigated the incidence of cardiovascular disease including coronary artery disease and stroke.

**Results:**

Over a median follow-up period of 8.7 years, 871 of 7728 (11.3%) subjects developed incident CKD. Patients with prediabetes, as defined by IGT or HbA1_C_, developed incident CKD more frequently than the non-prediabetic group did. The risk of CKD development at follow-up was analyzed according to different prediabetes definitions. Compared with the non-prediabetic group, the IGT- (Hazard ratio [HR] = 1.135, 95% confidence interval [CI] = 1.182–1.310, *P* = 0.043) and HbA1_C_-defined prediabetic groups (HR = 1.391, 95% CI = 1.213–1.595, *P* < 0.001) were significantly associated with incident CKD after adjusting for traditional CKD risk factors; however, IFG was not associated with incident CKD.

**Conclusion:**

IGT- or HbA1_C_-defined prediabetes is an independent predictor of incident CKD. The measurement of these parameters might enable early detection of CKD risk, allowing physicians to initiate preventive measures and improve patient outcomes.

## Background

The prevalence of chronic kidney disease (CKD), which is characterized by structural kidney damage and chronic reduction of kidney function, has increased globally with an increase in the aging population [1]. CKD constitutes a major health problem because it increases all-cause mortality and the development of cardiovascular disease (CVD) [2]. Therefore, it is important to identify modifiable risk factors that can act as early predictors of incident CKD.

Diabetes is the leading cause of CKD in most countries, and up to one-third of adults with newly diagnosed diabetes already have CKD, suggesting that CKD may occur in the early stages of diabetes [3, 4]. Prediabetes, which is regarded as a CVD risk factor, is an intermediate state of hyperglycemia, wherein blood glucose is higher than normal but lower than that in diabetes [5, 6]. Considering that prediabetes can be a precursor of diabetes, we hypothesized that prediabetes is associated with incident CKD, through the effect of hyperglycemia. However, very few studies have investigated such an association. In addition, prediabetes can be defined according to impaired fasting glucose (IFG), impaired glucose tolerance (IGT), or HbA1_C_ level, and a previous study suggested that each of these components might have a different association with CKD [7].

The aim of the present study was to clarify the clinical impact of prediabetes, defined by IFG, IGT, and HbA1_C_ level, on the development of incident CKD in a Korean adult population using data from the Korea Genome and Epidemiology Study (KoGES).

## Methods

### Study population

The study included all individuals who participated in the Ansung–Ansan cohort study from 2001 to 2002 (baseline) to 2011–2012 (fifth follow-up visit). The Ansung–Ansan cohort study is an ongoing study that began in 2001 and involves biennial follow-up examinations. A total of 10,038 patients were initially enrolled in the cohort. The design and baseline characteristics of the Ansung–Ansan cohort study have been previously published [8]. The exclusion criteria were as follows: pre-existing CKD, diabetes, lack of follow up, and insufficient data. Informed consent was obtained from all study subjects. The study protocol was approved by the ethics committee of the Korean Centre for Disease Control.

### Clinical and biochemical parameters

Study data included medical history, physical examination findings, information collected using a questionnaire, anthropometric measurements, and laboratory measurements. A standard questionnaire was used to obtain information on medical history, family history, current medication use, weekly alcohol consumption, and smoking status from all participants.

Waist circumference was measured three times using a fiberglass tape measure at the midpoint between the bottom of the ribcage and the top of the iliac crest. Blood pressure was measured using a standard mercury sphygmomanometer with the subject in the sitting position after 5 min of rest.

Body mass index (BMI) was calculated as weight divided by height squared (kg/m^2^). Physical activity was classified into the following three categories: none, irregular (≤2 episodes/week), and regular (≥3 episodes/week) exercise. One episode of exercise was defined as exercising for at least 30 min. Subjects were classified as “generally obese” if their BMI was ≥25 kg/m^2^. Collected blood samples were delivered to and analyzed at a central laboratory (Seoul Clinical Laboratories, Seoul, Korea). Plasma glucose, total cholesterol, triglycerides, and high-density lipoprotein cholesterol levels were determined using a Hitachi 747 chemistry analyzer (Hitachi, Tokyo, Japan). The low-density lipoprotein cholesterol level was calculated using Friedewald’s equation. The HbA1_C_ level was measured using high-performance liquid chromatography with a Variant II instrument (BioRad Laboratories, Hercules, CA, USA).

### Definitions of prediabetes cardiovascular disease and CKD

Prediabetes was defined as the satisfaction of at least one of the following three conditions: 1) fasting plasma glucose level of 110–125 mg/dl (IFG), 2) glucose levels of 140–199 mg/dL (7.8–1.0 mmol) on the 75-g oral glucose tolerance (IGT) 2 h after glucose consumption, or 3) HbA1_C_ concentration of 5.7–6.4%. Estimated glomerular filtration rate (eGFR) was calculated using the CKD Epidemiology Collaboration equation (CKD-EPI). According to the National Kidney Foundation Kidney Disease Outcomes Quality Initiative, CKD was defined as an eGFR of < 60 mL/min/1.73 m^2^. After an overnight-fast, the subjects had a 75-g glucose challenge, and blood samples were collected at 0, 60, and 120 min for oral glucose tolerance test (OGTT). OGTT test was supervised and conducted by trained nurse. Serum glucose concentrations and HbA_1c_ were measured using an automatic analyser (ZEUS 9.9; Takeda, Tokyo, Japan). The incidence of cardiovascular disease (CVD) (included coronary artery disease, stroke, peripheral artery disease) was defined as at least one positive response to CVD-related questionnaire items, including diagnosis by a physician, treatment, or use of medications.

### Statistical analyses

Data are expressed as mean and standard deviation or as the number of subjects and percentage. Baseline variables were compared according to the presence or absence of CKD by using Student’s t test for continuous variables and the χ^2^ test for categorical variables. We calculated the hazard ratios (HRs) for incident CKD by using Cox proportional hazards models with potential confounding variables. All analyses were performed using SPSS Statistics for Windows version 18.0 (IBM, Armonk, NY, USA). For all tests, a *P* value < 0.05 was considered to indicate a statistically significant difference.

## Results

The mean age of subjects was 52 years, with 52.6% of subjects being male. Mean follow-up duration was 8.7 years. Among the 10,038 patients, 7728 patients were included in this study, after excluding 216 patients with CKD, 1046 with diabetes, 982 patients who were never followed up, and 66 patients with insufficient data.

The anthropometric and biochemical characteristics of the subjects are summarized in Table 1 according to the development of incident CKD. Traditional risk factors for CKD such as older age, obesity, hypertension, and metabolic syndrome were more prevalent in the CKD group. Prediabetes defined according to IGT or HbA1_C_ level was more frequently noted in the CKD group, but the incidence of IFG was not different between the CKD and non-CKD groups. Interestingly, male sex and regular exercise were associated with a lower incidence of CKD. The incidence of CVD according to IGT is shown in Table 2. The incidence of diabetes and CKD increased in subjects with IGT. The incidence of other CVDs, including stroke and coronary artery disease, did not differ according to IGT. Using a Cox proportional hazards model, we also investigated the clinical impact of IGT on the development of CKD during the follow-up period. Participants with IGT had a higher risk for CKD development (hazard ratio [HR] 1.14, 95% confidence interval [CI] 1.182–1.310, *P* = 0.043), as shown in Table 3. Moreover, this significant association persisted after adjusting for other CKD risk factors, including age, sex, obesity, hypertension, and metabolic syndrome. Traditional CKD risk factors such as age, obesity, hypertension, and metabolic syndrome also significantly increased the development of CKD after adjusting for confounding factors. We also analyzed the effect of other prediabetes definitions on CKD development by using a Cox model. Consistent with the IGT findings, prediabetes defined by HbA1_C_ level was also significantly associated with CKD development after adjusting for possible confounding factors (HR 1.39, 95% CI 1.21–1.60, *P* < 0.001) as shown in Table 4. IFG was not associated with future CKD development.

**Table 1:** Characteristics of the subjects according to incident chronic kidney disease

	Newly developed CKD (*n* = 871)	No CKD (*n* = 6857)	*P*-value
Age (years)	57 ± 8	51 ± 8	0.001
Male	610 (70.0)	3456 (50.4)	< 0.001
Body mass index > 25 kg/m^2^	421 (48.3)	2737 (39.9)	< 0.001
Hypertension	332 (38.1)	1487 (21.7)	< 0.001
Current Smoking	137 (15.7)	1351 (19.7)	0.041
Regular exercise	499 (57.3)	4198 (61.2)	0.027
Metabolic syndrome	227 (26.1)	1147 (16.7)	< 0.001
Fasting glucose, mg/dl	87.7 ± 9.0	87.9 ± 8.9	0.592
Impaired fasting glucose	84 (9.6)	652 (9.5)	0.902
Impaired glucose tolerance	297 (34.1)	1882 (27.4)	< 0.001
5.7 < HbA1_C_ < 6.5	425 (48.8)	2461 (35.9)	< 0.001
Low density lipoprotein, mg/dl	117 ± 33	113 ± 32	< 0.001
Triglyceride, mg/dl	172 ± 105	153 ± 95	< 0.001
High-density lipoprotein, mg/dl	44 ± 10	45 ± 10	< 0.001
AST, IU/L	29 ± 18	29 ± 16	0.921
ALT, IU/L	26 ± 23	27 ± 21	0.036
Creatinine (mg/dL)	0.87 ± 0.18	0.82 ± 0.16	< 0.001
eGFR (mL/min/1.73 m^2^)	85.2 ± 12.2	93.5 ± 15.1	< 0.001
Follow-up duration	9.0 ± 2.1	8.7 ± 2.5	< 0.001

*CKD* chronic kidney disease, *AST* aspartate aminotransferase, *ALT* alanine aminotransferase, *eGFR* estimated glomerular filtration rate

Data are presented as mean ± standard deviation or n (%)

**Table 2:** Incidence of chronic kidney disease and other cardiovascular diseases according to impaired glucose tolerance and HbA1c defined prediabetes

	IGT (+) (*n* = 2179)	IGT (−) (*n* = 5549)	*P*-value	HbA1_C_ 5.7–6.4% (*n* = 2886)	HbA1_C_ < 5.7% (*n* = 4842)	*P*-value
CKD	297 (14.1)	574 (10.6)	< 0.001	425 (14.7)	446 (9.2)	< 0.001
Hypertension	694 (31.8)	1862 (33.6)	0.155	984 (34.1)	1572 (32.5)	0.147
Diabetes	479 (22.0)	280 (5.0)	< 0.001	573 (19.9)	186 (3.8)	< 0.001
CAD	65 (3.0)	131 (2.4)	0.126	65 (2.3)	88 (1.8)	0.205
Stroke	47 (2.2)	88 (1.6)	0.100	64 (2.2)	71 (1.5)	0.019
PAD	3 (0.1)	8 (0.1)	1.0	4 (0.1)	7 (0.1)	1.0

*CAD* coronary artery disease, *CKD* chronic kidney disease, *IGT* impaired glucose tolerance, *PAD* peripheral artery disease

Data are presented as n (%)

**Table 3:** Hazard ratio of chronic kidney disease according to prediabetes, body composition, and metabolic syndrome component

	Non-adjusted		Adjusted	
	Odds ratio (95% confidence interval)	*P*-value	Odds ratio (95% confidence interval)	*P*-value
IGT	1.362 (1.184–1.567)	< 0.001	^a^1.135 (1.182–1.310)	0.043
Prediabetes defined by HbA1_C_	1.661 (1.454–1.897)	< 0.001	^b^1.391 (1.213–1.595)	< 0.001
Baseline eGFR (60–89 vs. ≥90)	4.704 (3.499–4.744)	< 0.001	^b^3.727 (3.184–4.363)	< 0.001
Male sex	2.136 (1.848–2.469)	< 0.001	^b^2.310 (1.993–2.77)	< 0.001
Age > 65 years	3.347 (2.878–3.892)	< 0.001	^b^1.918 (1.631–2.255)	< 0.001
Current smoking	1.121 (0.841–1.212)	0.242		
BMI > 25 kg/m^2^	1.347 (1.180–1.539)	< 0.001	^b^1.100 (0.954–1.269)	0.190
Hypertension	2.104 (1.835–2.412)	< 0.001	^b^1.606 (1.375–1.875)	< 0.001
Regular activity	0.853 (0.745–0.975)	0.020	^b^0.859 (0.751–0.983)	0.027
Metabolic syndrome	1.318 (1.167–1.632)	0.012	^b^1.125 (0.943–1.341)	0.191

*BMI* body mass index, *eGFR* estimated glomerular filtration, *IGT* impaired glucose tolerance

^a^multivariate analysis was done including IGT, baseline eGFR, age, BMI, hypertension, regular activity, metabolic syndrome

^b^multivariate analysis was done including prediabetes defined by HbA1_C_, baseline eGFR, age, BMI, hypertension, regular activity, metabolic syndrome

**Table 4:** Hazard ratio of development of chronic kidney disease according to prediabetes component

	IGT	*P*-value	IFG	*P*-value	HbA1_C_	*P*-value
Crude hazard ratio	1.362 (1.184–1.567)	< 0.001	1.023 (0.817–1.281)	0.843	1.661 (1.454–1.897)	< 0.001
Model 1	1.270 (1.104–1.462)	0.001	1.157 (0.923–1.451)	0.206	1.299 (1.135–1.486)	< 0.001
Model 2	1.135 (1.182–1.310)	0.043	0.918 (0.721–1.168)	0.485	1.391 (1.213–1.595)	< 0.001

*IGT* impaired glucose tolerance, *IFG* impaired fasting glucose

Model 1 adjusted for age > 65 and sex

Model 2 adjusted for age > 65, sex, hypertension, obesity, regular activity, baseline eGFR (60–89 vs. ≥90) and metabolic syndrome

## Discussion

In this large prospective community-based cohort study of Korean adults, we found that prediabetes defined by IGT or HbA1_C_ was significantly associated with the development of CKD, independent of traditional CKD risk factors. However, the incidence and relative risk for CKD was not associated with IFG. To the best of our knowledge, this is the first and largest population-based prospective study to evaluate the association between prediabetes, as assessed by IGT, HbA1_C_, and IFG, and the development of CKD.

In addition, older age (> 65 years), BMI, hypertension, and metabolic syndrome were revealed as risk factors for CKD in our community-based cohort. These results were in line with previous studies. Older age [9], obesity [10], hypertension [11], and metabolic syndrome [12] are well-recognized risk factors for developing CKD. Other risk factors, including dyslipidemia and hypertension are also associated with the development of CKD in patients with diabetes [13, 14]. Male gender was associated with an increased risk of CKD in our study. Previous studies showed the prevalence of CKD is higher in women than in men including US renal data system (USRDS) annual data report [15–17]. However, there were also opposing data regarding this issue [18–20]. Korea data also showed female gender was associated with less reduced renal function (Odds ratio 0.874, 95% CI 0.766–.0.997) and trend toward lower incidence of CKD (Odds ratio 0.953, 95% CI 0.848–1.071) [21]. Thus, we think that there might be a geographic variability in the effect of gender on the prevalence of CKD.

Several studies have shown that prediabetes and metabolic syndrome are associated with CKD development, after adjustment for established CKD risk factors [22–26]. In one meta-analysis that included 9 cohort studies (8 IFG-defined prediabetes and 1 HbA_1C_-defined prediabetes study) showed that the overall relative risk of CKD was 1.12 (95% CI. 1.02–1.21), after adjustments for established risk factors [7]. However, most of the studies defined prediabetes according to IFG, since IGT determination requires 2-h post-glucose measurement. Our study is different from earlier studies that reported an association between prediabetes and CKD because we used three different definitions of prediabetes. Interestingly, there was no difference in baseline fasting glucose level or the incidence of IFG according to the presence of overt CKD in our subjects. In contrast, baseline IGT- or HbA1_C_-defined prediabetes was more prevalent in the CKD group, and the hazard ratio was also robustly higher after adjusting for traditional CKD risk factors. It is difficult to know exactly whether this is an incidental finding or a meaningful difference. In previous studies, only one definition was studied, or both IGT and IFG were included in the definition of prediabetes [26]. Although, both IGT and IFG are included in prediabetes, the clinical impact or underlying mechanism might be different. We think IGT or HbA1_C_ level may better reflect long-term exposure to non-diabetic hyperglycemia than IFG. Previous study reported that IFG reflects defects in insulin secretion and IGT reflects insulin resistance [27]. Milicevic et al. [28] reported that IGT and HbA1_C_ were more strongly associated with cardiovascular outcomes than IFG was. Therefore, clinical significance of each indicator is expected to be studied in the future.

Previous researchers suggested some potential mechanisms by which prediabetes might affect the initiation and progression of CKD. Individuals with prediabetes can be as insulin-resistant as individuals with diabetes. Thus, dysfunction in the metabolism of lipids and carbohydrates and loss of protein stores, which are often observed in patients with insulin-resistance, may be associated with kidney damage in prediabetic individuals [29, 30]. Cross-sectional studies have revealed that borderline hyperglycemia or prehypertension is associated with glomerular hyperfiltration, which is a marker of early renal damage [31, 32]. Thus, in subjects with prediabetes or prehypertension, especially with comorbid glomerular hyperfiltration, earlier treatment of hyperglycemia or high blood pressure may be necessary to prevent the development of kidney damage. In addition, insulin resistance may lead to CKD by alteration of endothelial function and inflammation, sodium retention, and activation of the sympathetic nervous system [33, 34].

The present study has several advantages over previous studies. As mentioned above, we examined all the three commonly used prediabetes definition parameters (IFG, IGT, and HbA1_C_) to identify their relationship with CKD. Second, this study prospectively followed a relatively large number of subjects for an average of 9 years. Development of incidental CKD is preceded by glomerular hyperfiltration, which is an important link between prediabetes and CKD development. Thus, short or intermediate follow-up periods might not capture the association between prediabetes and CKD. Moreover, although most of the published studies have relied on GFR estimated using the Modification of Diet in Renal Disease Study (MDRD) equation [7], we defined CKD using the CKD-EPI equation, which performs better than the MDRD equation, especially at higher GFR, with less bias and greater accuracy [35]. Because most individuals with prediabetes have higher GFR, the CKD-EPI equation might better reflect kidney function.

This study has several limitations. First, GFR was not measured directly, but was estimated using a serum creatinine-based equation, which might have overestimated or underestimated the actual GFR. Second, we did not have information on urine analysis including microalbuminuria, a known risk factor for the development of kidney disease in individuals with diabetes. Considering CKD is defined by the presence of albuminuria as well as by eGFR, overall CKD may be underestimated in this study. Third, because all participants were required to attend follow-up examination, those who died or developed serious comorbidities were excluded; thus, we may have introduced survival bias. Fourth, this study was restricted to only Koreans. Therefore, these results might not be applicable to other ethnic groups.

## Conclusions

The results of this study show that subjects with prediabetes defined by IGT or HbA1_C_ level have an increased risk of incident CKD; however, IFG was not associated with CKD. Thus, our findings indicate that IGT or HbA1C are predictors of CKD, and might enable clinicians to better prevent the development of CKD in individuals with prediabetes, potentially resulting in better outcomes.

## Abbreviations

BMI: Body mass index
CKD: Chronic kidney disease
CVD: Cardiovascular disease
eGFR: Estimated glomerular filtration rate
IFG: Impaired fasting glucose
IGT: Impaired glucose tolerance
